# Factors Influencing Practice of Pesticide Use and Acute Health Symptoms among Farmers in Nakhon Sawan, Thailand

**DOI:** 10.3390/ijerph18168803

**Published:** 2021-08-20

**Authors:** Teera Kangkhetkron, Chudchawal Juntarawijit

**Affiliations:** 1 Department of Natural Resources and Environment, Faculty of Agriculture Natural Resources and Environment, Naresuan University, Phitsanulok 65000, Thailand; teera2727@hotmail.co.th; 2 Nakhon Sawan Provincial Public Health Office, Ministry of Public Health, Nakhon Sawan 60000, Thailand

**Keywords:** knowledge, attitude and practice, pesticide handling practice, pesticide exposure, farmer health, acute health symptoms

## Abstract

Information on knowledge (K), attitude (A), and practice (P) in terms of pesticide use is essential for an effective exposure control program. The objectives of this study were to survey the level of knowledge, attitude, and practice in terms of pesticide use, and the prevalence of acute health symptoms (AHSs) among farmers in Nakhon Sawan Province, Thailand. The study also tried to identify factors affecting the practice of pesticide use. Data from 680 farmers were collected using a face-to-face interview questionnaire. The relationship between safety practices and related factors was analyzed using ordinal logistic regression. This study found about 40% of the farmers had a good level of practice. Factors affecting practice were education, work experience, level of knowledge, or attitudes. Many participants experienced acute health symptoms in the past 24 h, and these symptoms were significantly associated with poor practice (*p* < 0.05). Public health organizations should provide farmers with more information, especially on chronic effects of pesticides.

## 1. Introduction

Agriculture is the second-largest sector in the world with regard to workforce, with over one billion workers. It is one of the most dangerous occupations, with 250 million accidents every year and 170,000 deaths [1]. Pesticide exposure is the issue of greatest concern among farmworkers. Most agricultural activities, e.g., mixing and spraying pesticides, harvest, and cultivation are usually performed by hand, causing farmworkers to be exposed to chemical substances in agriculture [2]. Pesticides have been linked with both acute and chronic health effects. The World Health Organization and United Nations Environmental Program have estimated that there will be up to five million cases of pesticide poisoning among agricultural workers each year and that will include about 20,000 death cases [3,4]. Pesticide exposure has been associated by several studies with various kinds of chronic health consequences, e.g., cancer [5,6,7,8], amyotrophic lateral scleral [9], asthma [10], diabetes [11], leukemia [12], Alzheimer’s, and Parkinson’s disease [13,14].

Poor practice of pesticide use could increase the risk of pesticide exposure and the abovementioned risks. A study among farmers in the Ecuadorian Amazon basin reported an association between poor practice and inadequate protection during pesticide application with acute intoxication [15]. In Thailand, however, pesticides are intensively used without effective and adequate protection practice [16]. The Thai National Statistical Office reported around 32% of the working population (12.37 million) are working in agriculture [17]. This group of workers usually disproportionately comes from lower socioeconomic strata in Thai society [16]. Data from the Ministry of Public Health showed that cases of pesticide intoxication were from 76.4 to 96.6 per 100,000 people [18]. Studies among rice farmers in Sukhothai Province, Thailand found that the frequency, concentration, and duration of pesticide use had a strong association with acute health symptoms [19].

It was found that practice (P) is linearly associated with knowledge (K) and attitude (A) [20,21]. A survey among 220 farmers in Pathum Thani Province found a good level of knowledge (70.1%), attitudes (69.6%), and prevention behaviors (69.5) [22]. Another study among rice farmers in Chainart Province reported that the majority of farmers had moderate levels of knowledge (74.5%) and attitude (81.6%), but a poor level of practice (78.5%) [23]. A study among farmers in Ubonrachathani Province found that farmers had a low level of knowledge (77.2%), and attitude (54.5%), but fair practices (85.0%). Additionally, a study on the association between practice and knowledge (r = 0.285) and practice and attitude (r = 0.305) revealed a small positive correlation [24].

For AHSs, a study among rice farmers in Chainart Province reported 33.7% as having minor symptoms, e.g., headache, fatigue, dizziness, stomach cramps, and throat irritation, and 13.3% as having moderate signs, e.g., pupil contraction, excessive sweating, and excessive secretion of saliva [23]. Another study among rice farmers from northern Thailand reported a high prevalence of dry throat and cramp, and these symptoms among those conducted spraying and mixing activities [24].

To date, there has been no study on the issues among farmers in Nakhon Sawan Province, Thailand. This study aimed to survey the level of knowledge, attitudes, and practices of pesticide use among farmers in the province, and to identify factors affecting the safe practice of pesticide use. The study also tried to associate levels of practice and prevalence of AHSs. The finding of this study will be useful for exposure prevention and training programs for safe pesticide use.

## 2. Materials and Methods

### 2.1. Study Design, Setting, and Site

This descriptive cross-sectional study was conducted in Nakhon Sawan Province, Thailand. Nakhon Sawan Province is located in the central region, about 250 km north of Bangkok, with a population of 1,066,455 people and 401,432 households in its 15 districts (data from 2016). The majority of inhabitants are farmers, and the main crops include rice, sugarcane, and cassava. In 2014, the province had a gross domestic product (GDP) of THB 21,852 (USD 716). Approximately 78% of the land in this province is used for agriculture, and there are 308,430 agricultural workers [25,26,27].

### 2.2. Study Participants and Sampling Procedure

In this study, farmers referred to agricultural workers who worked in the production of crops such as rice, cassava, sugarcane, corn, vegetables, etc. Study participants were farmers aged 20 years and older, who had worked as farmers for at least three years. The participants were selected from all 15 districts in Nakhon Sawan. In each district, a subdistrict was randomly selected by a lottery method. In each subdistrict, 0.23% of farmers were randomly selected by village health volunteers working in a local hospital using systemic random sampling.

The sampling frame for the selection of farmers was obtained from the Nakhon Sawan Provincial Agriculture and Cooperatives Office of the agriculture workers registered in Nakhon Sawan Province [27].

The minimum sample size was estimated according to Krejcie and Morgan [28] with the following formula and assumptions:

n = [χ^2^NP(1 − P)]/[d^2^ (N − 1) + χ^2^P(1 − P)]

where:

n = the sample size
N = the total number of crop-growing farmers in Nakhon Sawan, which was 308,430
χ^2^ = chi-square for the specified confidence level at 1 degree of freedom or desired confidence level 0.05 = 3.841
P = percentage of farmers using pesticides on their farm, which was assumed to be 0.4 [29]
d = denotes margin of error, degree of accuracy expressed as a proportion of 0.05 at a 95% confidence level.

Therefore, using the formula n = [3.841 × 308,430 × 0.4 × (1 − 0.4)]/[(0.05)^2^ × (308,430 − 1) + 3.841 × 0.4 × (1 − 0.4)] = 345.8, adding design effect = 345.8 × 2 = 691.6, and assuming a non-response rate of 4%, the total sample size estimated for the study was 719.

### 2.3. Questionnaire, Study Variables, and Scoring

The questionnaire consisted of several close-ended questions. It was tested for content validity, yielding an index of item objective congruence (IOC) of 0.67–1.00. The questionnaire also underwent pilot testing for reliability with 30 farmers who had similar characteristics to the participants. Corrections and adjustments were made to any ambiguous questions. KR-21 was used to assess the reliability of knowledge of pesticides, yielding a value of 0.93. Cronbach’s alpha coefficients for internal consistency reliability were 0.89 for attitude about pesticide use and 0.88 for practice of pesticide use.

The questionnaire had three major parts, as follows:

Part 1 included demographic data about gender, age, education completed, and pesticide use.

Part 2 included KAP with questions about knowledge, attitude, and practice in terms of pesticide use. The knowledge section consisted of 14 items (Items 1 to 14) about possible routes of entry for pesticides, problems related to pesticide exposure, and the health effects of long-term exposure to pesticides. The items were rated on a yes or no scale, scoring 0 or 1 where 1 was given for each correct answer. The highest possible total score was 14.

The level of knowledge was classified into high level with a score of 80% (≥11.2 points), moderate level with a score of 61–79% (8.4–11.1 points), and low level with a score of 60% (<8.4 points) [30].

The attitude about using pesticides, proper use of personal protective equipment (PPE), and safe work procedures when handling the pesticide were assessed with items 1 to 8. Items were rated on a three-point scale of 1 (disagree), 2 (not sure), and 3 (agree). The highest possible total score was 24.

The level of attitude was classified into concerned level with a score of 80% (≥19.2 points), neutral level with a score of 61–79% (14.5–19.1 points), and not concerned level with a score of 60% (<14.5 points).

The practice of pesticide use, safety precautions to be taken when using pesticide, and good labeling and storage of pesticide was evaluated with items 1 to 12. Items were rated on a three-point scale of 3 (always), 2 (sometimes), and 1 (rarely). The highest possible total score was 36.

The level of practice was classified into a good level with a score of 80% (≥28.8 points), a fair level with a score of 61–79% (21.7–28.7 points), and a poor level with a score of 60% (<21.7 points).

The classification of total score of knowledge, attitude, and practice was adapted from the study by Bloom [30].

Part 3 assessed the farmer’s acute health symptoms after using pesticides in the past 24 h, including difficulty breathing, rash/ulcer/blister, irritated eyes, nausea/vomiting, unconsciousness, and headache. Each farmer could choose one or more acute symptoms.

### 2.4. Data Collection

Data were collected by village health volunteers (VHVs). The VHVs are volunteers who work with the local hospital. This group has a major role in health promotion and disease prevention in the community. They were trained to support public health officers in the communication of health information, data collection, and providing basic care to patients with chronic diseases. Currently, they are paid and receive health benefits from the government [31]. All 15 local hospitals in the selected districts were contacted and 75 membership village health volunteers (VHVs) were invited to participate in the study. In this study, only VHVs who had mobile phones and access to online questionnaires were recruited using simple random sampling. To ensure consistency across interviews, the VHVs and the local public health officer also had to attend a one-day training session about the research and were trained for interviewing the participants, along with the correct use of online questionnaires. Most of the interviews took place at the participant’s home, but sometimes in other places (e.g., local temple or hospital). Data were collected between 1 October 2019 and 31 December 2020.

### 2.5. Statistical Analysis

Data from all 680 participants were included in data analysis. Collected data were analyzed using IBM SPSS Statistics (version 25) and STATA (version 14.2). The *p*-values of <0.05 were considered statistically significant. Demographic data were analyzed using descriptive statistics (i.e., frequency, percentage, mean, median, and standard deviation).

The associations of demographic characteristics, knowledge, and attitude with practice of pesticide exposure were determined using ordinal logistic regression presented as a crude odds ratio (OR), and 95% confidence intervals (CIs) were compared with people in the low-level group as a reference.

The associations between education, duration of pesticide use, knowledge, attitude, practice and acute health symptoms were determined using ordinal logistic regression presented as a crude odds ratio (OR), adjusted odds ratio (AOR), and 95% confidence intervals (CIs), compared with people in the high-level group as a reference and adjusted ORs for gender (male, female) and age (≤54, 55–64, 65–74, ≥75).

### 2.6. Ethical Considerations

This study was approved by the Ethics Board of Naresuan University (project number 550/60). Written informed consent for the interviews was obtained from each subject before the interviewing process.

## 3. Results

### 3.1. Demographic Information

In this study, 680 of the 719 sampled farmers participated (participation rate = 94.6%). Male farmers accounted for 59.0% of the participants. Most of the farmers were between 55 and 74 years old (61.6%), with a mean age of 65.6 years. The highest education was primary school (Grade 1–6) (91.0%). Regarding pesticide use, the majority of farmers had used pesticides for over 20 years (64.4%), with a mean duration of pesticide use of 24.29 years (Table 1).

### 3.2. Level of Knowledge, Attitude, and Practice

The results showed that 37.9% of farmers had a high level of knowledge. A small proportion of them thought that pesticide toxicity was related to chronic diseases, e.g., diabetes (32.7%), asthma (33.0%), and Alzheimer’s/Parkinson’s disease (38.5%). For attitude, 51.2% of farmers had a concerned level of attitude. They had an unconcerned attitude about the statement that using many pesticides at the same time can save time when spraying pesticides and can be carried out without any concerns (74.7%), and that using a higher concentration than recommended could increase effectiveness of the pesticides (70.0%). Regarding practice, about 40% had a good level of practice overall. Most farmers read the label and followed the instruction as recommended (68.4%), and 58.1% wore PPE while spraying or working with pesticides (Table 2 and Table A1, Table A2 and Table A3).

### 3.3. Factors Influencing Practice

The farmers with secondary school education (Grade 7–12) and higher had a better practice (OR = 1.77, 95% CI = 1.07–2.93) than those who only finished primary school (Table 3). This study also found a better practice among those with more working experience. For knowledge, the farmers had high knowledge had good practice (OR = 5.58, 95% CI = 2.96–9.87). Similarly, concern and attitude were correlated with good practice (OR = 4.99, 95% CI = 2.82–8.85) (Table 4).

### 3.4. Farmers’ Acute Health Symptoms

It was found that many of the farmers experienced acute health symptoms after handling pesticides during the previous 24 h (Table 5). The most common symptoms were difficulty breathing (47.2%), nausea/vomiting (46.9%), diarrhea (42.8%), and rash (42.1%). Prevalence of the symptoms was highly correlated with poor practice of pesticide use (OR = 2.17, 95% CI = 1.08–4.35) (Table 6).

## 4. Discussion

### 4.1. Levels of Knowledge, Attitude, and Practice

The study found only 37.9% of farmers had a high level of knowledge. Many of them did not know that pesticide exposure might cause chronic diseases, e.g., diabetes (32.7%), asthma (33.0%), and Alzheimer’s/Parkinson’s disease (38.5%). This was supported by a previous study in Thailand that also found poor knowledge among rice, flower, and vegetable farmers in Nakornratchasima, Phisanulok, and Payao Province [33]. Similar results were also observed in Ethiopia which found only 23.6% of farmers know that pesticide can cause a chronic disease [34]. Public health organizations should provide farmers with more information, especially on the chronic health effects of pesticide use.

This study found that 51.2% of the farmers had a concerned level in attitude evaluation. Meanwhile, 18.4% of them were not concerned that using many pesticides at the same time could affect pesticide efficiency and 20.6% were not concerned about the problem of using a higher concentration than recommended. A previous study by Norkaew (2010) also found that 12.1% of farmers strongly agreed that using more pesticides than indicated on the label might increase yield [24]. A study in Nepal reported that only 16.9% of farmers read the label about pesticide toxicity [35].

In terms of proper farming practice, only 39.8% of the participants had a good score (Table 2). However, a closer look at individual questions in Appendix C (Table A3) showed that a large proportion of farmers had a high score on PPE and other protective clothing while spraying or working with pesticides. This result was consistent with previous research in Ubonrachathani Province reporting that 63.6% of farmers wore PPE or other ‘protective’ clothing [24]. However, data on the appropriateness and types of PPE in use are lacking and questionable due to problems relating to methods of data collection and analysis. Previous studies reported that Thai farmers usually wrapped a cotton cloth around their faces instead of wearing a chemical face mask that can trap pesticide spray [36]. This was consistent with another study that reported farmers wearing long trousers (56%), long-sleeved shirts (75%), a cloth wrapped around their face (74%), cotton gloves (34%), a balaclava (39%), and a disposable paper mask (35%) [33].

### 4.2. Predictive Factors of Practice

Further analysis indicated a higher level of practice among farmers who completed secondary school and higher (OR = 1.77, 95% CI = 1.07–2.93) or those having working experience of over ten years (OR = 1.49, 95% CI = 1.26–1.95) (Table 3). This was consistent with a study in Nakhon Nayok Province, Thailand which also found good practice among long-term pesticide users [37]. Levels of practice also significantly increase among the farmers with high levels of knowledge (OR = 1.76, 95% CI = 1.03–3.02), concern (OR = 1.76, 95% CI = 1.03–3.02), and a neutral level of attitude (OR = 1.76, 95% CI = 1.03–3.02) (Table 4). This result was supported by previous studies in other settings in Thailand, e.g., among farmers in Nakhon Nayok Province [37], Pathum Thani Province [22], Ubonrachathani Province [24], and Chainart Province [23]. A recent study in Nepal also found a significant association between knowledge and practice (*p* < 0.001) [35]. A similar result was also found in a study in Malaysia [38].

### 4.3. Acute Health Symptoms (AHSs)

In this study, the farmers experienced many of the surveyed AHSs, such as difficulty breathing (47.2%), nausea/vomiting (46.9%), diarrhea (42.8%), and rash (42.1%). This result was supported by data from the Disease Control Department of Thailand which reported the prevalence of pesticide poisoning from farming as 13.54 per 100,000 people, mostly related to the use of organophosphates and carbamate insecticides and herbicides [39]. This figure was underestimated and did not include those with less severe cases which did not require hospitalization [25].

This high prevalence of AHSs was also reported in other studies in Thailand [10]. Studies among farmers in other provinces in the same region reported 46.5% with cough, 44.2% with nasal congestion, 30.2% with chest tightness [33], numbness (41.2%), irritated eyes (42.9%), difficulty breathing/chest pain (37.9%), dizziness (34.6%), and fatigue (33.5%) [25]. In Malaysia, excessive sweating (34.7%), hand and leg numbness (34.0%), blurry vision (27.1%), headache (22.9%), and coughs (22.9%) were recorded [38]. A study in the Gaza Strip reported eye/face irritation in 64.3%, dizziness (32.4%), and difficulty in breathing/chest pain (28.1%) [40]. In Indonesia, the most frequent symptoms were fatigue (60%), muscle stiffness (54%), and dry throat (30%) [41]. However, a lower was rate found in Nepal, with the prevalence of dizziness and headache being 11.1% and that of skin allergy being 9.9% [35].

The prevalence of AHSs was significantly associated with poor practice (OR = 2.17, 95% CI = 1.04–4.35) (Table 6). This supports the notion that poor practice causes greater exposure, leading to further consequences. Similar results were also observed in farmers in Sukhothai Province, Thailand [19], Nepal [35], Tanzania [42], and Iran [43]. 

### 4.4. Limitation and Bias

This study may have some limitations. The study result may represent farmers in Nakhon Sawan, Thailand only. Associations with other groups of farmers should be carried out with caution. Data on acute health symptoms experienced by the farmers might be subject to recall bias although the symptoms were limited to those occurring in the past 12 months [44,45]. The major strength of this study was that it collected data from large groups of farmers and the relative associations could be estimated. The results could be used for developing projects and policies for the prevention of pesticide exposure and protection of farmers’ health.

## 5. Conclusions

Most of the farmers in Nakhon Sawan Province had a poor-to-fair practice of pesticide use. About half of them had a low level of knowledge and a concerned level of attitude. The levels of practices were significantly associated with education level, durations of pesticide use, levels of knowledge, and attitudes. However, we did not find associations with gender and age. Many of them experienced acute symptoms in the past 12 months and the prevalence of the symptoms was significantly associated with poor practice. Public health organizations should provide farmers with more information to reduce pesticide exposure, especially among inexperienced farmers.

## Figures and Tables

**Table 1:** Demographic data of participating farmers in Nakhon Sawan (N = 680).

Characteristic	Frequency	Percent
Gender		
Male	401	59.0
Female	279	41.0
Age		
≤54	105	15.4
55–64	200	29.5
65–74	218	32.1
≥75	157	23.0
Mean ± SD	65.64 ± 12.45	
Median (min–max)	65.00 (31–98)	
Education		
Primary school (Grade 1–6)	619	91.0
Secondary school (Grade 7–12) and higher	61	9.0
Duration of pesticide use (years)		
≤5	66	9.7
6–10	102	15.0
11–20	74	10.9
Mean (years) ± SD	24.29 ± 14.29	
Median (min–max)	26.00 (3–60)	

**Table 2:** Distribution of scores for knowledge, attitude, and practice among farmers in Nakhon Sawan (N = 680).

Factors/Variables	Level/Score	Number	Percent
Knowledge	High (11.2–14.0)	258	37.9
	Moderate (8.4–11.1)	97	14.3
	Low (<8.4)	325	47.8
Attitude	Concerned (19.2–24.0)	348	51.2
	Neutral (14.5–19.1)	276	40.6
	Not concerned (<14.5)	56	8.2
Practice	Good (28.8–36.0)	271	39.8
	Fair (28.7–21.7)	333	49.0
	Poor (<21.7)	76	11.2

**Table 3:** Associations between demographic and practice level (N = 680).

Factors	Practice Level						OR (95% CI) ^1^
	Poor		Fair		Good		
	N	%	N	%	N	%	
Gender							
Female	31	11.1	140	50.2	108	38.7	Reference
Male	45	11.2	193	48.1	163	40.7	1.06 (0.79–1.42)
*p* for trend							0.720
Age (years)							
≤54	12	11.4	55	52.4	38	36.2	Reference
55–64	22	11.0	105	52.5	73	36.5	1.03 (0.65–1.61)
65–74	22	10.1	104	47.7	92	42.2	1.25 (0.80–1.95)
≥75	21	13.4	68	43.3	68	43.3	1.20 (0.75–1.94)
*p* for trend							0.375
Education							
Primary school (Grade 1–6)	74	12.0	305	49.3	240	38.7	Reference
Secondary school (Grade 7–12) and higher	2	3.3	28	45.9	31	50.8	1.77 (1.07–2.93)
*p* for trend *							0.048
Pesticide use (years)							
≤5	11	16.7	24	36.4	31	46.9	Reference
6–10	8	7.8	48	47.1	46	45.1	1.13 (0.61–2.07)
11–20	11	14.9	45	60.8	18	24.3	1.49 (1.26–1.95)
>20	46	10.5	216	49.3	176	40.2	1.90 (1.54–1.92)
*p* for trend *							0.012

^1^ Crude odds ratio of ordinal logistic regression; statistically significant at *p* < 0.05. * *p*-values forlinear trends were derived using a continuous variable with midpoint of each category.

**Table 4:** Associations between knowledge, attitude, and practice level (N = 680).

Factors	Practice Level						OR (95% CI) ^1^
	Poor		Fair		Good		
	N	%	N	%	N	%	
Knowledge							
High	8	3.1	46	17.8	204	79.1	5.58 (2.96–9.87)
Moderate	18	18.6	62	63.9	17	17.5	0.96 (0.62–1.54)
Low	50	15.4	225	69.2	50	15.4	Reference
*p* for trend *							<0.001
Attitude							
Concerned	20	5.7	159	45.7	169	48.6	4.99 (2.82–8.85)
Neutral	39	14.1	146	52.9	91	33.0	2.45 (1.38–4.36)
Not concerned	17	30.3	28	50.0	11	19.7	Reference
*p* for trend *							<0.001

^1^ Crude odds ratio of ordinal logistic regression statistically significant at *p* < 0.05. * *p*-values forlinear trends were derived using a continuous variable with midpoint of each category.

**Table 5:** Prevalence of acute health symptoms among farmers in Nakhon Sawan (N = 680).

Health Symptom *	Frequency	Percent
Respiratory		
Difficulty in breathing	321	47.2
Chest pain	212	31.5
Epithelial/mucosal surfaces		
Rash	286	42.1
Irritated eyes	274	40.3
Ulcer/blister	142	20.9
Gastrointestinal		
Nausea/vomiting	317	46.9
Diarrhea	291	42.8
Stomach pain	275	40.4
Other		
Unconscious	108	15.9
Headache	91	13.4

* Classification of acute pesticide poisoning [32].

**Table 6:** Predictive factors of acute health symptoms among farmers in Nakhon Sawan (N = 680).

Factors	Acute Health Symptoms		
	Yes (N, (%))	OR (95% CI) ^1^	AOR (95% CI) **
Practice			
Good	198 (37.6)	Reference	Reference
Fair	264 (50.1)	1.41(0.96–2.05)	1.37(0.93–2.01)
Poor	65 (12.3)	2.17(1.08–4.35)	2.21(1.10–4.44)
*p* for trend *		0.035	0.007

^1^ Crude odds ratio of ordinal logistic regression with statistically significance at *p* < 0.05. * *p*-values forlinear trends were derived using a continuous variable with midpoint of each category. ** Logistic regression adjusted for gender (male, female), age (≤54, 55–64, 65–74, ≥75).

## Data Availability

The data are not publicly available due to privacy.

## Appendix A

**Table A1:** Knowledge of pesticide use and its potential health effects among farmers in Nakhon Sawan (N = 680).

Knowledge Description	Agree		Disagree	
	N	%	N	%
Pesticides can enter the body by…				
Inhalation	662	97.3	18	2.7
Ingestion	664	97.6	16	2.4
Skin absorption	648	95.3	32	4.7
Pesticides can affect humans, animals, and all other living organisms	652	95.9	28	4.1
Pesticides can affect environment and ecological systems.	651	95.7	29	4.3
Pesticides are associated with…				
Cancer	522	76.8	158	23.2
Skin and subcutaneous tissue diseases	388	57.0	292	43.0
Allergy	355	52.2	325	47.8
Asthma	225	33.0	455	67.0
Fetal maturity	361	53.1	319	46.9
Child’s developmental delay	354	52.1	326	47.9
Fertility and infertility	324	47.6	356	52.4
Alzheimer’s disease/Parkinson’s disease	262	38.5	418	61.5
Diabetes	223	32.8	457	67.2

## Appendix B

**Table A2:** Attitude about pesticide use, and exposure prevention among farmers in Nakhon Sawan (N = 680).

Attitude Description	Agree		Not sure		Disagree	
	N	%	N	%	N	%
We should take a bath right after spraying pesticides.	622	91.5	34	5.0	24	3.5
We should avoid entering the field or workplace after spraying pesticides.	618	90.9	36	5.3	26	3.8
While spraying or working with pesticides, we should avoid eating, drinking, and smoking.	589	86.6	40	5.9	51	7.5
Wearing boots is essential to prevent harmful effects while spraying pesticides.	615	90.4	35	5.2	30	4.4
Using personal protective equipment (PPE) is necessary and worth buying.	187	27.5	465	68.4	28	4.1
Pesticides can leave residues in agricultural products and affect consumers.	180	26.5	432	63.5	68	10.0
Using many pesticides at the same time can save time when spraying pesticides and can be done without any concerns.	125	18.4	47	6.9	508	74.7
Using a higher concentration than recommended could increase effectiveness of the pesticides.	140	20.6	64	9.4	476	70.0

## Appendix C

**Table A3:** Pesticide use practices among farmers in Nakhon Sawan (N = 680).

Practice Description	Always		Sometimes		Rarely	
	N	%	N	%	N	%
Before spraying						
I read the label and follow the instruction as recommended.	465	68.4	97	14.3	118	17.3
Keep pesticides in special storage outside the house.	282	41.5	273	40.1	125	18.4
Spraying						
Do not smoke while spraying or working with pesticides.	277	40.7	140	20.6	263	38.7
Do not eat or drink while spraying or working with pesticides.	267	39.3	202	29.7	211	31.0
Wear appropriate PPE while spraying or working with pesticides.	395	58.1	158	23.2	127	18.7
Wear boots while spraying or working with pesticides.	358	52.6	157	23.1	165	24.3
Wear a protective mask while spraying or working with pesticides.	320	47.1	187	27.5	173	25.4
Wear gloves while spraying or working with pesticides.	416	61.2	182	26.7	82	12.1
Wear a long sleeve shirt and long trousers while spraying or working with pesticides.	335	49.3	205	30.1	140	20.6
After spraying						
Change contaminated clothes and shower immediately after spraying.	298	43.8	125	18.4	257	37.8
Wash working clothes right after spraying, and separate from other clothes.	284	41.8	219	32.2	177	26.0
Dispose of pesticide containers separately from general solid waste.	276	40.6	278	40.9	126	18.5
